# WILDbase: towards a common database to improve wildlife disease surveillance in Europe

**DOI:** 10.2807/1560-7917.ES.2024.29.25.2300617

**Published:** 2024-06-20

**Authors:** Marieke P de Cock, Valérie O Baede, Sara R Wijburg, Sara A Burt, Robert FNA van Tiel, Kim K Wiskerke, Jens RJ van der Post, Wim HM van der Poel, Hein Sprong, Miriam Maas

**Affiliations:** 1 National Institute for Public Health and the Environment, Centre for Infectious Disease Control, Bilthoven, The Netherlands; 2 Wageningen University and Research, Quantitative Veterinary Epidemiology, Wageningen, The Netherlands; 3 Utrecht University, Institute for Risk Assessment Science (IRAS), Utrecht, The Netherlands; 4 Wageningen Bioveterinary Research, Lelystad, The Netherlands

**Keywords:** Public health, Epidemiology, Rodent, Oryctolagus cuniculus, Erinaceus europaeus, Talpa europaea, Martes foina, Vulpes vulpes, wildlife database

## Abstract

**Background:**

To be better prepared for emerging wildlife-borne zoonoses, we need to strengthen wildlife disease surveillance.

**Aim:**

The aim of this study was to create a topical overview of zoonotic pathogens in wildlife species to identify knowledge gaps and opportunities for improvement of wildlife disease surveillance.

**Methods:**

We created a database, which is based on a systematic literature review in Embase focused on zoonotic pathogens in 10 common urban wildlife mammals in Europe, namely brown rats, house mice, wood mice, common voles, red squirrels, European rabbits, European hedgehogs, European moles, stone martens and red foxes. In total, we retrieved 6,305 unique articles of which 882 were included.

**Results:**

In total, 186 zoonotic pathogen species were described, including 90 bacteria, 42 helminths, 19 protozoa, 22 viruses and 15 fungi. Most of these pathogens were only studied in one single animal species. Even considering that some pathogens are relatively species-specific, many European countries have no (accessible) data on zoonotic pathogens in these relevant animal species. We used the Netherlands as an example to show how this database can be used by other countries to identify wildlife disease surveillance gaps on a national level. Only 4% of all potential host–pathogen combinations have been studied in the Netherlands.

**Conclusions:**

This database comprises a comprehensive overview that can guide future research on wildlife-borne zoonotic diseases both on a European and national scale. Sharing and expanding this database provides a solid starting point for future European-wide collaborations to improve wildlife disease surveillance.

## Introduction

More than 60% of all emerging infectious diseases are zoonotic and of those, more than 70% originate from wildlife [1]. Emerging wildlife diseases are a result of spillover events between wildlife and humans [2], and they seem to be becoming more frequent, often due to increased contact rates between humans and wild animals, in part driven by habitat loss and/or modification, increased urbanisation, changes in agricultural practices and the globalisation of trade and travel [3-5], as well as shifting ranges of wildlife and vectors due to climate change [6]. The health, economic and societal consequences of zoonotic spillover events from wildlife can be far-reaching, as exemplified by the recent COVID-19 pandemic [7-10].

Urban areas can be considered artificial ecosystems that provide ample opportunity for zoonotic pathogen spillover between wildlife, domestic animals and humans, and they can therefore form hotspots of increased zoonotic disease risk [11,12]. This is due to the close contact between humans and wildlife and the altered wildlife composition in urban areas compared with nature areas, which could result in new spillover events between wildlife species [2,5,13]. This in turn could lead to different pathogen transmission cycles and disease dynamics in urban areas and consequently to higher rates of exposure to zoonotic pathogens [14-16]. Moreover, changes to the urban environment that promote biodiversity, such as urban greening [17-20], could further alter disease dynamics in urban areas. These complex interactions underline the importance of broadening wildlife disease surveillance to multi-host and multi-pathogen communities to increase our understanding of pathogen transmission cycles in urban areas and to identify potential pathogen spillover between wildlife species. However, surveillance of wildlife diseases in urban areas can be challenging and most wildlife studies are performed outside of urban areas [21-23]. To facilitate wildlife surveillance in urban areas, having a comprehensive overview of zoonotic pathogens studied in urban wildlife species to determine how to set up this surveillance will help prioritise monitoring efforts.

While there are many studies investigating zoonotic pathogens in wildlife species, there is no common database where this information is systematically collected and stored. This study aimed to improve the surveillance and cross-country collaboration of wildlife-borne zoonoses, especially for, but not limited to, urban areas, by creating such a comprehensive and easily accessible database. To this end, we performed a systematic literature review of zoonotic pathogens studied in 10 common urban wildlife mammals. We present and summarise knowledge gaps in the monitoring of wildlife-borne zoonotic pathogens in Europe per pathogen, animal species, country, and habitat type. Further, we used the Netherlands as an example to demonstrate how this database can be used by other countries to identify their current knowledge gaps in wildlife disease surveillance. These knowledge gaps deserve more attention to identify potential pathogen spillover between species or the presence of multiple competent host species. The complete database underlying this study, named *WILDbase*, is publicly available at https://wildbase.org and in Supplement 1.

## Methods

In this review we considered zoonotic pathogens comprising bacteria, viruses, helminths (including Annelida, Platyhelminthes, Nematoda and Acanthocephala), unicellular eukaryotes (hereafter called protozoa, and including Apicomplexan parasites) and fungi. Ectoparasites were excluded. This review was not registered beforehand.

### Selection of wildlife species

We selected 10 common urban wildlife mammal species, based on their presence in Dutch cities. The majority of these species also occurs in most other European countries; for a distribution across countries, we refer to the appended Supplementary Figure S1. For the selection, we used data from the Dutch National database for Flora and Fauna for six of the larger Dutch cities (Amsterdam, Utrecht, Nijmegen, Eindhoven, Maastricht and Enschede) in combination with expert opinions from urban ecologists (Jan Buijs, Floris Brekelmans, Ton Verhoeven, Bart ter Beek, Frank Verhagen and René Janssen). Species were selected that were present in areas with varying levels of urbanicity of all six cities. This led to the following list of 10 common urban mammal species (excluding flying mammals, i.e. bats), in random order: brown rat (*Rattus norvegicus*), house mouse (*Mus musculus*), wood mouse (*Apodemus sylvaticus*), common vole (*Microtus arvalis*), red squirrel *(Sciurus vulgaris*), European rabbit (*Oryctolagus cuniculus*), European hedgehog (*Erinaceus europaeus*), European mole (*Talpa europaea*), stone marten (*Martes foina*) and red fox (*Vulpes vulpes*).

### Search strategy

We conducted a systematic literature search according to the PRISMA guidelines to identify articles, however using only the Embase database [24]. Search terms included keywords related to (i) the Latin species name and any equivalent English names, (ii) grammatical variations of the term ‘zoonosis’ and (iii) a list of 86 important emerging zoonotic pathogens compiled by order of the Dutch government [25,26]. A combination of search terms (i) AND (ii) and (i) AND (iii) was used. The search terms included wildcards to capture term variations (e.g., zoono*). The search terms used per animal species can be found in Supplementary Table S1. We included all articles published before 1 January 2023, which resulted in a total of 6,305 unique articles. Some of these described multiple species of interest and were thus screened more than once, summing up to 7,403 species-specific reports to be screened.

### Article screening and exclusion

**Figure 1:**
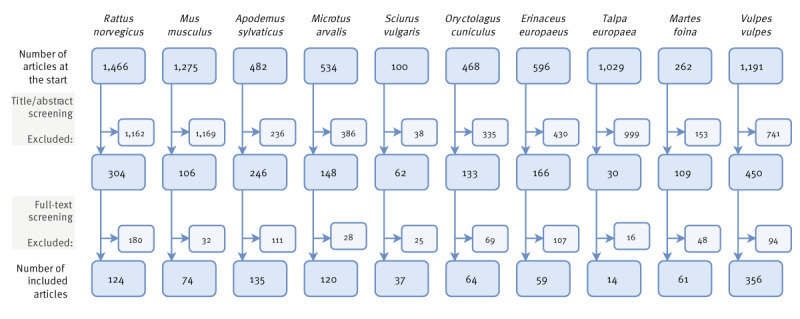
Overview of the article screening process by animal species, wildlife disease surveillance, Europe, up to 1 January 2023 (n = 7,403^a^)

^a^ Shown are the numbers of reports for each species. As some species were covered in the same publication, there were a total of 7,403 species-specific reports covered in 6,305 unique articles.

Per species, we first screened the title and abstract, which excluded 5,649 of 7,403 reports, followed by a full-text screening, which excluded 710 of 1,754 species-specific reports. All species summed up, this resulted in 1,044 species-specific reports, covered in 882 unique articles, that were used for data extraction (Figure 1). All articles were assessed by two independent reviewers (MdC and VB/JvdP/RvT/KW), or three (MM/SB/VB) in case of disagreement between the first two reviewers. Consensus was sought in case of disagreement. For studies to be included, they needed to research: (i) one of the 10 mammal species of interest, (ii) wild animals (i.e. not pet or laboratory animals) and (iii) zoonotic pathogens tested in those animals. A pathogen species was considered zoonotic when we could find at least one case of human infection with the pathogen in scientific literature. In case a pathogen was only identified to genus level but not to species level, information on zoonotic potential could often not be determined. Therefore, a pathogen genus was considered ‘zoonotic’ when a zoonotic species belonging to that genus had been detected at least once in the same animal species in Europe. A pathogen genus was considered ‘potentially zoonotic’ when no specific zoonotic species belonging to that genus had been detected in the same animal species in Europe up to 2023, although that genus may contain zoonotic species. Exclusion criteria included: (i) the research was performed outside of Europe (a list of included European countries can be found in Supplementary Table S2), (ii) the article did not contain original data (e.g. a review or modelling study using published data), (iii) species-specific data could not be extracted from the article (e.g., different rodent species were grouped together and only the total prevalence of that group was reported), (iv) the article was not peer-reviewed (e.g. a preprint or conference abstract), (v) the full article was written in a language other than English, (vi) it was a duplicated article and (vii) the full text was not available (mainly the case for older articles). The article screening process is summarised in Figure 1.

### Data extraction and processing

We extracted the following data from the articles: pathogen species (or genus, in case the pathogen was not determined to species level), tissue(s) sampled, laboratory methods used, mammal species studied, total number of animals tested, number of animals testing positive, country, publication year and habitat type of trapping. Habitat type was classified into urban (including peri- and suburban areas), rural, agriculture (including animal farms, crop farms and fields), nature, or other (e.g., wild animals in an animal rescue centre). This classification was made based on the habitat information provided in the articles. When animals were trapped in multiple habitat types, but the results were not reported separately per habitat type, we classified the habitat type as ‘mixed’. In case no or too few details were given about the habitat type to be classified with certainty into one of the categories, we classified the habitat type as ‘unspecified’. Some older articles contained obsolete country names (e.g., Yugoslavia), which we changed to current country names by matching them with the exact trapping locations.

To determine the completeness of wildlife disease surveillance on a European or national level, we calculated the ‘coverage’ by dividing the number of studied host–pathogen combinations by the total number of possible host–pathogen combinations, multiplied by 100. The distribution of animals in Europe was retrieved from the International Union for Conservation of Nature’s Red List of Threatened Species website [27]. We used R version 4.3.1 (21 September 2023) for the creation of distribution maps [28].

## Results

### Data overview

**Figure 2:**
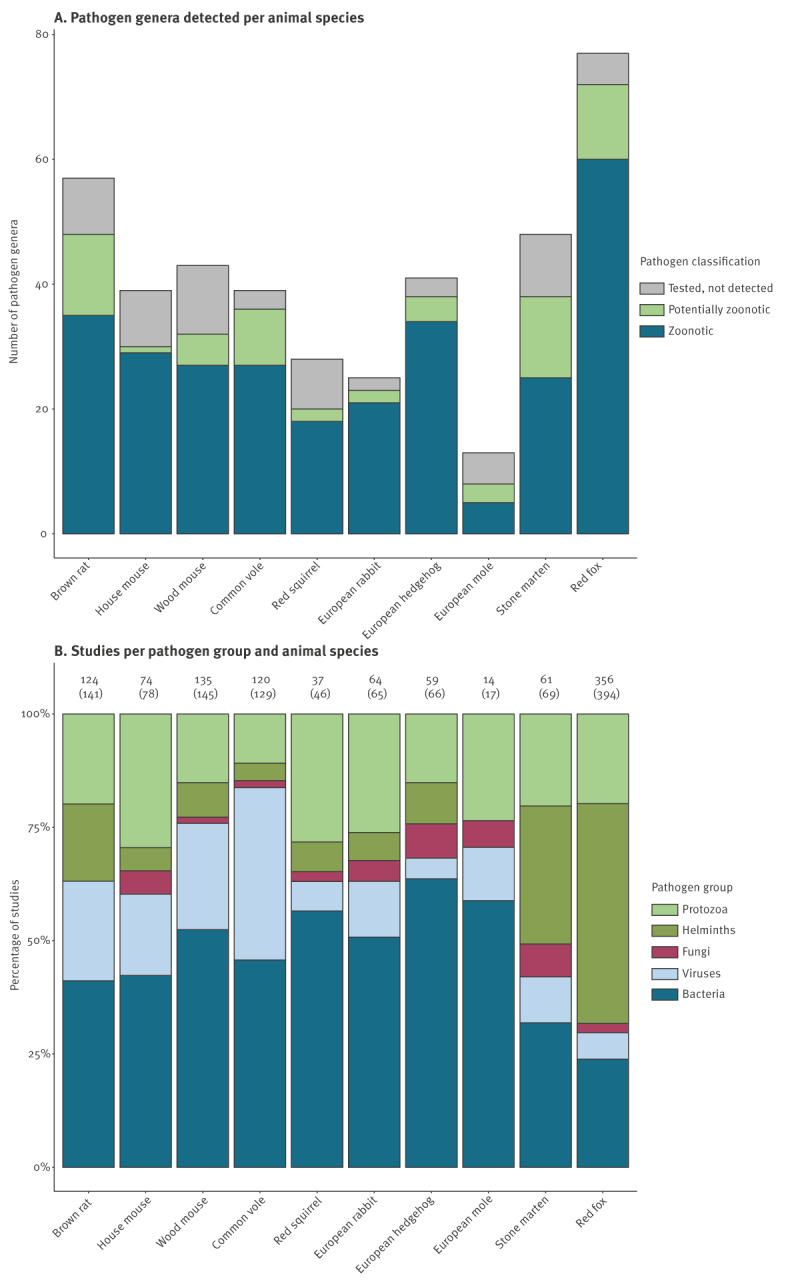
Wildlife disease surveillance of (potentially) zoonotic pathogens in different wildlife species, Europe, up to 1 January 2023 (n = 882)

In total, we included 882 articles providing data on 102 zoonotic pathogen genera, 21 potentially zoonotic pathogen genera, and 186 zoonotic pathogen species. This database is accessible at https://wildbase.org and is also appended as Supplement 1. Supplementary Table S3 gives an overview of all studied zoonotic pathogens in the 10 selected wildlife species and visualises all current knowledge gaps per pathogen group, genus and species, and per animal species. As an example, we provide here a shortened version of this table focusing only on viruses (Table). Most studied pathogens belonged to the group of bacteria (44 genera, 90 species and 363 studies), followed by helminths (37 genera, 42 species and 254 studies), protozoa (11 genera, 19 species and 182 studies), viruses (20 genera, 22 species and 151 studies) and lastly fungi (11 genera, 15 species and 27 studies) (Table, Figure 1, Figure 2).

**Table:** Overview of studied (potentially) zoonotic viruses in different wildlife species, Europe, up to 1 January 2023 (n = 151 studies)

Viral pathogens		Animals											Number of animal species with the virus detected
Genus	Virus species or name	Order	*Rodentia*					*Lagomorpha*	*Eulipotyphla*		*Carnivora*		
		Family	*Muridae*			*Cricetidae*	*Sciuridae*	*Leporidae*	*Erinaceidae*	*Talpidae*	*Mustelidae*	*Canidae*	
		Animal species	Brown rat	House mouse	Wood mouse	Common vole	Red squirrel	European rabbit	European hedgehog	European mole	Stone marten	Red fox	
*Alphainfluenzavirus*	*Influenza A virus*		0/1^a^	−	−	−	−	−	0/1^a^	−	**1/2^a^**	**3/4**	2
*Alphavirus*	*Sindbis virus*		−	−	−	−	−	−	**1/1^a^**	−	−	−	1^a^
*Aphthovirus*	*Foot-and-mouth disease virus*		−	−	−	−	−	−	**1/1^a^**	−	−	−	1^a^
*Betacoronavirus*	*SARS-CoV-2*		0/1^a^	0/1^a^	0/2^a^	0/1^a^	−	−	−	−	**1/2**	0/2^a^	1
*Cardiovirus*	*Encephalomyocarditis virus*		0/1^a^	−	−	−	−	−	−	−	−	−	0^a^
*Coltivirus*	*Eyach coltivirus*		−	−	0/1	−	−	0/1^a^	−	−	−	−	0
*Flavivirus*	spp		0/1^a^	−	0/1^a^	−	−	−	−	−	−	−	0^a^
	*Tick-borne encephalitis virus*		−	0/1^a^	**2/4^b^**	**4/4**	0/1^a^	−	**1/1^a^**	**2/2^a^**	0/1^a^	**1/2^a^**	5
	*Usutu virus*		0/1^a^	−	−	−	−	−	−	−	−	−	0^a^
	*West Nile virus*		0/1^a^	−	0/1^a^	−	−	−	**1/1^a^**	−	−	**1/2^a^**	2^a^
*Kobuvirus*	spp		**1/1^a^**	−	−	−	−	−	−	−	−	**1/1^a^**	2^a^
*Lyssavirus*	*European bat lyssavirus*		−	−	−	−	−	−	−	−	**1/1^a^**	−	1^a^
	*Lyssavirus rabies*		−	−	−	**1/2^a^**	−	−	−	−	**1/2^a^**	**3/8^a^**	3^a^
*Mammarenavirus*	*Lymphocytic choriomeningitis mammarenavirus*		−	**4/4^a^**	**5/6^a^**	**3/3^a^**	−	−	−	−	−	**1/1^a^**	4^a^
*Norovirus*	spp		**4/4^a^**	−	−	−	−	−	−	−	−	−	1^a^
*Orthobornavirus*	*Borna disease virus*		−	−	−	−	−	−	−	−	0/1^a^	**2/4^a^**	1^a^
*Orthohantavirus*	spp		**4/5^a^**	**2/2^a^**	**3/6^a^**	**5/7^a^**	−	−	−	−	−	**2/2^a^**	5^a^
	*Dobrava-Belgrade orthohantavirus*		−	0/1^a^	**2/8^a^**	**2/2^a^**	−	−	−	−	−	−	2^a^
	*Puumala orthohantavirus*		−	**2/3^a^**	**3/10^b^**	**1/3^a^**	−	−	−	−	−	**1/1^a^**	4
	*Seoul orthohantavirus*		**9/10**	0/1^a^	−	−	−	−	−	−	−	−	1
	*Tula orthohantavirus*		−	0/2^a^	**2/6^b^**	**25/26**	−	−	−	−	−	−	2
*Orthohepevirus*	spp		**1/1^a^**	−	−	**2/2^a^**	−	−	−	−	−	−	2^a^
	*Hepatitis E virus*		**10/12^a^**	0/1^a^	0/1^a^	−	−	**5/6**	−	−	−	**2/4^a^**	3
*Orthonairovirus*	*Crimean-Congo haemorrhagic fever virus*		−	−	0/1^a^	−	−	0/1^a^	−	−	−	−	0^a^
*Orthopoxvirus*	spp		0/1^a^	**1/1^a^**	**4/7^a^**	**2/2^a^**	−	−	−	−	−	−	3^a^
	*Cowpoxvirus*		**3/4**	**1/1^a^**	**3/3^a^**	**3/3^a^**	−	−	−	−	−	−	4
*Parechovirus*	*Ljungan virus*		−	**1/1^a^**	**1/1^a^**	−	**1/1^a^**	−	−	−	−	−	3^a^
*Phlebovirus*	spp		−	−	−	−	−	−	−	−	−	**1/1^a^**	1^a^
*Rotavirus*	spp		**2/2^a^**	−	−	−	0/1^a^	−	−	−	−	**2/2^a^**	2^a^
*Varicellovirus*	*Pseudorabies virus*		−	−	−	−	−	−	−	−	−	**1/1^a^**	1^a^
Number of viral pathogens detected			8	6^a^	9	10	1^a^	1	4^a^	1^a^	4	13	NA

CoV: coronavirus; NA: not applicable; SARS: severe acute respiratory syndrome.

− Host-pathogen combination not studied.

^a^ Host–pathogen combinations not studied in the Netherlands.

^b^ Host–pathogen combinations studied in the Netherlands but without any positive results.

Numbers are given as number of studies in which the pathogen was detected/total number of studies performed. Detected pathogens are shown in bold. Detailed results for bacteria, protozoa, helminths, and fungi are appended in Supplementary Table S3.

Panel A: All zoonotic and potentially zoonotic genera were also detected in the specific animal species. Panel B: The number above each bar shows the total number of studies per animal species and the number in brackets shows the total number of pathogens described in these studies per animal species.

### Coverage of the database

The total coverage for all host–pathogen combinations in this database was 26%, meaning that only a quarter of all potential host–pathogen combinations from Supplementary Table S3 have been studied in Europe so far. These coverages per species are appended in Supplementary Table S4 and ranged from 19% for helminths and fungi to 37% for protozoa. By animal species, coverage ranged from 7% for European moles to 57% for red foxes.

Most zoonotic pathogens (54%) were studied in only a single animal species. Only 11% of all pathogens (mostly bacteria and protozoa) were studied in five or more different animal species; the detailed numbers can be found in Supplementary Figure S2 and Supplementary Table S3. *Toxoplasma gondii* has been detected in all 10 animal species, which shows that multiple animal species can serve as (accidental) reservoir host. *Leishmania infantum*, *Francisella tularensis* and *Anaplasma phagocytophilum* have been detected in eight different animal species. In contrast, for example *Corynebacterium ulcerans*, *Proteus mirabilis*, *Streptococcus suis* and *Sindbis virus* have only been studied in one animal species.

In more than 60% of the studies, the type of environment (e.g., urban, rural, agriculture or nature) was not specified; we provide additional detail by animal species and habitat in Supplementary Figure S3. Only a few studies (< 10%) have been performed in urban areas and of those, almost half were conducted on brown rats. For example, *Cryptosporidium parvum*, *Taenia martis* and *Enterocytozoon bieneusi* have not been studied in urban areas.

### Differences between European countries; the Netherlands as an example

**Figure 3:**
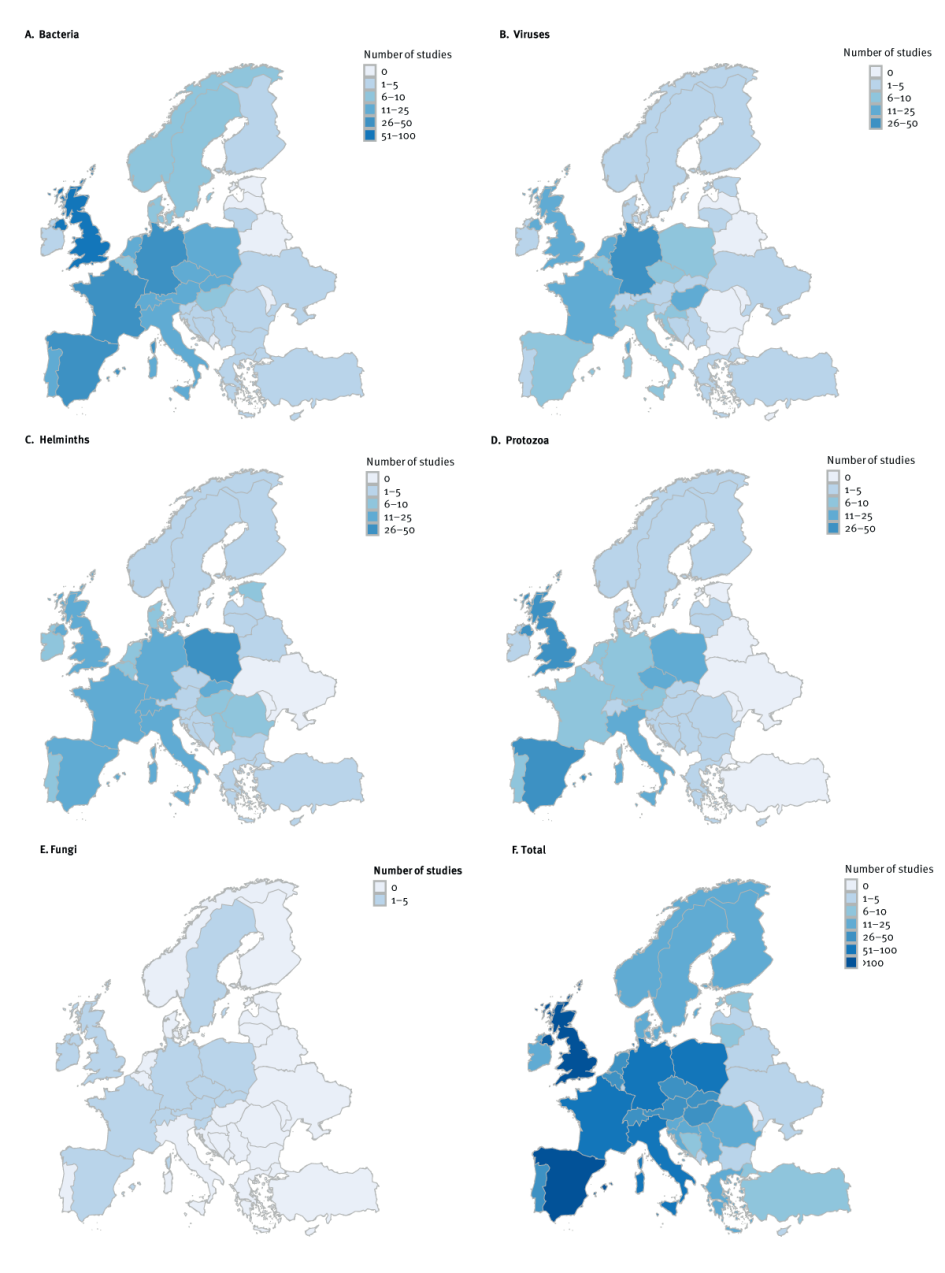
Number of studies conducted per pathogen group per country, Europe, up to 1 January 2023 (n = 882)

There were large differences in surveillance effort between European countries, both per pathogen group (Figure 3) and per animal species; for details on the number of animals tested per study see the additional data in Supplementary Figure S1. The total surveillance effort per country ranged from zero studies per country (for Albania, Kosovo^#^ and Moldova) to > 100 (for Spain and the United Kingdom) (Figure 3). To demonstrate how countries could use this database to identify their current knowledge gaps in wildlife disease surveillance, we marked the gaps for the Netherlands in the Table (footnote ^a^). This footnote indicates that while that specific pathogen has been studied in a specific animal species in Europe, none of those studies were performed in the Netherlands. The data for other pathogens can be found in Supplementary Table S3. As a result, even more knowledge gaps arose on a national level, and the overall coverage for all pathogens together decreased from 26% on a European level to only 4% for the Netherlands. 

## Discussion

This overview shows that the number of studies per pathogen group resembles their importance in causing zoonoses globally, except for viruses, which are in second place for zoonotic importance versus fourth place in this study [29]. This suggests that there is an underrepresentation of studies focusing on viruses in the included wildlife species in Europe.

Of all potential host–pathogen combinations in Supplementary Table S3, an average of 26% was covered by studies in our database. However, this coverage varied between pathogen group and animal species, which shows that there is a large surveillance bias, which hinders comparisons of the relative zoonotic disease risk posed by these different animal species [29]. For some of the non-studied host–pathogen combinations, the absence of research might be related to the current absence of the pathogen or its vector in a specific area due to environmental or climatic conditions [30-32] or to the pathogen being strictly associated with a single host [33]. However, pathogen detection is also sensitive to research effort and since the majority of pathogens can infect more than one host species [33], most of these pathogens have probably not been tested in all their potential host species yet, which may cause some poorly studied species to be misclassified as non-hosts. This highlights the importance of, and opportunities for, monitoring a broader spectrum of wildlife species to detect pathogen spillover and potentially new, unexpected reservoir hosts.

Urban and nature environments are very different in terms of animal species composition, abundance and richness [34], which may affect contact rates between animal species and pathogen transmission cycles. Despite the presumed increased zoonotic disease risks in urban areas due to increased contact between humans and animals [11,29,35], only a few studies (< 10%) have been performed in urban areas, with a focus on brown rats. Comparison of habitat types was complicated by the dependence on the habitat classification that was used in the article, which may have differed between studies. Also, in more than half of the studies, the type of environment was not specified, which hampers (risk) comparisons between environments. This highlights the need for studies to better specify where tested animals are from, to use standardised habitat descriptions and to increase the testing of animals in urban areas.

The differences in surveillance effort between European countries are probably due to country-specific variations in resource allocation for disease-related research [36], and to different perceived zoonotic risks from wildlife to humans. Comparing the coverage of host–pathogen combinations on a European and national scale for the example of the Netherlands, it became obvious that on a national level, there is an even lower coverage of host–pathogen combinations. It is important to increase this coverage to identify potential additional host reservoir species, better understand disease dynamics and prevent human infections. An example is tick-borne encephalitis virus (TBEV), an emerging zoonotic virus in the Netherlands [37]. Our overview shows that TBEV has already been detected in five different animal species, but we only tested for TBEV in two of those species in the Netherlands. In this way, TBEV could infect humans via other animal species than we might expect.

Deciding which knowledge gaps to address first (e.g. focusing on a specific pathogen or animal species) can be difficult and varies per country (Box).

Box **Suggested surveillance priorities**

1. Focus on host–pathogen combinations that have been detected in countries with similar environmental and climatic conditions;
2. Include host–pathogen combinations that have been previously tested, yet not detected, but for which the presence of a pathogen cannot be ruled out because there are few studies performed or few tested animals or because current knowledge of their host–pathogen potential is lacking;
3. Address the remaining host–pathogen combinations that have not been studied yet, starting with those pathogens that are likely to infect multiple animal species.

This decision can also be based on a risk assessment, as some pathogens pose a greater risk to human health than others, which can give additional directions for wildlife disease surveillance programmes. A risk assessment often includes parameters based on the (perceived) threat to human and/or animal health, which, among other things, depends on the pathogen’s prevalence in the animal population, the abundance of the animals, contact rates between humans and animals, pathogen transmission, infection prevalence in humans and disease symptoms in humans [25]. However, these parameters may be difficult to assess. For example, there is a lack of reliable data on animal abundance. Having a European database for wildlife abundance data, such as VectorNet for the abundance and distribution of vectors in Europe (https://www.ecdc.europa.eu/en/about-us/partnerships-and-networks/disease-and-laboratory-networks/vector-net), would be very useful. Besides that, the prevalence may vary depending on the test method used (e.g. PCR versus serology) and the type of tissue(s) tested (e.g. blood, organs or faeces), which may differ not only per study but also per pathogen. This complicates comparisons of relative zoonotic disease risk between pathogens.

In addition, the reliability of the observed prevalence is subject to the number of animals tested, which varied a lot between studies, from an average of 21 animals tested per study for European moles to 465 for red foxes, summarised in Supplementary Figure S4. This is partly related to the type of pathogen tested, the abundance of the animal species and access to the sample. For example, non-invasive faecal samples can be easier to collect and therefore result in higher sample sizes compared with organ or blood samples, which also often require ethical approval [38]. In addition, sample sizes may be very small especially for endangered or protected animal species. However, from the 10 animal species in this study, the European rabbit is the only one listed as ‘near threatened’ [27], while the average sample size of studies performed on European rabbits is larger than those performed on wood mice, brown rats, house mice, European hedgehogs, red squirrels, stone martens and European moles. A larger sample size will not only make the resulting prevalence more reliable, it also increases the chance of detecting pathogens that occur with low prevalence.

This systematic literature review resulted in a comprehensive, yet incomplete database of zoonotic pathogen research conducted in European wildlife species. One limitation is that we could have missed some relevant studies because our search was limited to the Embase literature database, or because of the search terms we used. For example, our search did not retrieve any studies about *Streptobacillus moniliformis* in wild brown rats in Europe, although brown rats are one of the main reservoir hosts [39]. All retrieved studies about *S. moniliformis* in brown rats were performed either outside Europe or on pet or laboratory rats. One relevant study had not been discovered in our search because ‘brown rat’, ‘*Rattus norvegicus*’ or an equivalent name from our search terms was not present in the title, abstract or keywords of that study [40]. A second limitation is that only a few studies reported negative results, probably a consequence of publication bias towards positive results [41]. However, the publication of negative results is very valuable and might enhance the coverage and accuracy of this database considerably. It is therefore important to extend this database with multiple literature databases and to have country-specific experts validate and enhance its accuracy.

While many studies have investigated zoonotic pathogens in wildlife species, our database shows that there is still a large surveillance bias regarding wildlife species and zoonotic pathogens, which results in knowledge gaps on a European level and even more on a national level. European-wide collaboration would improve zoonotic wildlife disease surveillance. A common topical database of published literature, for example an extension of WILDbase, preferably in combination with an online biobank for sharing animal samples, could identify and address current knowledge gaps, improve wildlife disease surveillance and justify or give direction to surveillance funding. As such, WILDbase would complement existing European networks such as EVD-Net (for emerging and vector-borne diseases) and EARS-Net (for antimicrobial resistance) [42]. In addition, such a database and biobank could result in more efficient use of animal samples (e.g., testing one sample for many pathogens), especially regarding ethical requirements related to invasive research on wildlife [38], the limited number of samples from protected or endangered species, and the time needed to collect and analyse the samples. When setting up such an extended database, usage of the vocabulary that is used in the Darwin Core model may facilitate integration with other databases [43].

## Conclusion

Increasing our knowledge about potential reservoir hosts for zoonotic pathogens cannot only help us to better understand pathogen transmission cycles and spillover, but will raise awareness among healthcare professionals, which may result in better identification of human disease cases. We encourage the usage and sharing of WILDbase (accessible at https://wildbase.org and in Supplement 1) by a broad range of European research institutes and professionals, as well as lay audiences. Expanding this comprehensive database (e.g., by consulting multiple literature databases or including more animal species) and keeping it up to date can provide a solid starting point for future European-wide collaborations to improve wildlife disease surveillance.

## ^#^Note

This designation is without prejudice to positions on status and is in line with United Nations Security Council Resolution 1244/99 and the International Court of Justice Opinion on the Kosovo Declaration of Independence.

## Supplementary Data

367000 Supplement1

## Supplementary Data

979000 Supplement2
