# Supplementary material for: WILDbase: towards a common database to improve wildlife disease surveillance in Europe
Source: Euro Surveill. 2024 Jun 20;29(25):2300617. doi: 10.2807/1560-7917.ES.2024.29.25.2300617 (PMC11191416; doi:10.2807/1560-7917.ES.2024.29.25.2300617)
Supplement: Supplement2 [file 23-00617_MAAS_SupplementaryTablesAndFigures.pdf]

Supplement

This supplementary material is hosted by Eurosurveillance as supporting information alongside the article ‘WILDbase: towards a common database to improve wildlife disease surveillance in Europe’, on behalf of the authors, who remain responsible for the accuracy and appropriateness of the content. The same standards for ethics, copyright, attributions and permissions as for the article apply. Supplements are not edited by Eurosurveillance and the journal is not responsible for the maintenance of any links or email addresses provided therein.

Table S1 Search terms per animal species. Per species, a combination of search terms (i) and (ii), and of search terms (i) and (iii) was used.

**(i) Species name**	**Brown rat**	'rattus norvegicus*':ti,ab,kw OR 'brown rat*':ti,ab,kw OR 'norway rat*':ti,ab,kw OR 'norwegian rat*':ti,ab,kw OR 'common rat*':ti,ab,kw OR 'street rat*':ti,ab,kw OR 'sewer rat*':ti,ab,kw OR 'hannover rat*':ti,ab,kw OR 'wharf rat*':ti,ab,kw OR 'tunnel rat*':ti,ab,kw OR 'gutter rat*':ti,ab,kw OR 'rattus norvegicus'/exp OR 'brown rat'/exp OR 'norway rat'/exp OR 'norwegian rat'/exp OR 'common rat' OR 'street rat' OR 'sewer rat' OR 'hannover rat' OR 'wharf rat' OR 'tunnel rat' OR 'gutter rat' OR 'urban rat*':ti,ab,kw OR 'urban rat'
	**House mouse**	'house mouse*':ti,ab,kw OR 'house mice*':ti,ab,kw OR 'mus musculus*':ti,ab,kw OR 'house mouse'/exp OR 'house mice' OR 'mus musculus'/exp OR 'mus domesticus*':ti,ab,kw OR 'mus domesticus'/exp
	**Wood mouse**	'wood mouse*':ti,ab,kw OR 'apodemus sylvaticus*':ti,ab,kw OR 'field mouse*':ti,ab,kw OR 'wood mouse'/exp OR 'apodemus sylvaticus'/exp OR 'field mouse' OR 'wood mice':ti,ab,kw OR 'field mice':ti,ab,kw OR 'wood mice' OR 'field mice'
	**Common vole**	'common vole*':ti,ab,kw OR 'microtus arvalis*':ti,ab,kw OR 'field mouse*':ti,ab,kw OR 'field mice*':ti,ab,kw OR 'common vole' OR 'microtus arvalis'/exp OR 'field mouse' OR 'field mice' OR ‘field vole’
	**Red squirrel**	'red squirrel*':ti,ab,kw OR 'sciurus vulgaris*':ti,ab,kw OR 'red squirrel' OR 'sciurus vulgaris'/exp
	**European rabbit**	'oryctolagus cuniculus*':ti,ab,kw OR 'european rabbit*':ti,ab,kw OR 'oryctolagus cuniculus'/exp OR 'european rabbit'/exp OR 'wild rabbit*':ti,ab,kw OR 'wild rabbit'
	**European hedgehog**	'hedgehog*':ab,ti,kw OR 'erinaceus europaeus*':ab,ti,kw OR 'hedgehog'/exp OR 'erinaceus europaeus'/exp
	**European mole**	'mole':ti,ab,kw OR 'talpa europaea*':ti,ab,kw OR 'moles':ti,ab,kw OR 'mole'/exp OR 'talpa europaea'/exp OR 'moles'/exp
	**Stone marten**	'marten*':ti,ab,kw OR 'beech marten*':ti,ab,kw OR 'martes foina*':ti,ab,kw OR 'stone marten*':ti,ab,kw OR 'house marten*':ti,ab,kw OR 'white breasted marten*':ti,ab,kw OR 'marten'/exp OR 'beech marten'/exp OR 'martes foina'/exp OR 'stone marten'/exp OR 'house marten'/exp OR 'white breasted marten'
	**Red fox**	'red fox*':ti,ab,kw OR 'vulpes vulpes*':ti,ab,kw OR 'red fox'/exp OR 'vulpes vulpes'/exp
**(ii) Emtree terms for ‘zoonosis’**	'zoono*':ti,ab,kw OR 'zoonosis'/exp OR 'zoonotic'	
**(iii) Emerging zoonoses list**	'ehrlichi*':ti,ab,kw OR 'ehrlichia'/exp OR 'bartonell*':ti,ab,kw OR 'bartonella'/exp OR 'brucell*':ti,ab,kw OR 'brucella'/exp OR 'burkholderia*':ti,ab,kw OR 'burkholderia'/exp OR 'chlamydophil*':ti,ab,kw OR 'chlamydophila'/exp OR 'clostridi*':ti,ab,kw OR 'clostridium'/exp OR 'coxiell*':ti,ab,kw OR 'coxiella burnetii'/exp OR 'anaplasm*':ti,ab,kw OR 'anaplasma'/exp OR 'escherichia coli':ti,ab,kw OR 'escherichia coli'/exp OR 'salmonell*':ti,ab,kw OR 'salmonella'/exp OR 'yersin*':ti,ab,kw OR 'yersinia'/exp OR 'erysipelothrix':ti,ab,kw OR 'erysipelothrix'/exp OR 'capnocytophag*':ti,ab,kw OR 'capnocytophaga'/exp OR 'francisella':ti,ab,kw OR 'francisella'/exp OR 'tularem*':ti,ab,kw OR 'tularemia'/exp OR 'leptospir*':ti,ab,kw OR 'leptospira'/exp OR 'weil s disease':ti,ab,kw OR 'weil s disease'/exp OR 'mycobacteri*':ti,ab,kw OR 'mycobacterium'/exp OR 'pasteurell*':ti,ab,kw OR 'pasteurella'/exp OR 'rickettsi*':ti,ab,kw OR 'rickettsia'/exp OR 'borreli*':ti,ab,kw OR 'borrelia'/exp OR 'staphylococc*':ti,ab,kw OR 'staphylococcus'/exp OR	

	'streptococc*':ti,ab,kw OR 'streptococcus'/exp OR 'campylobacter*':ti,ab,kw OR 'campylobacter'/exp OR 'cryptococc*':ti,ab,kw OR 'cryptococcus'/exp OR 'ascari*':ti,ab,kw OR 'ascaris'/exp OR 'baylisascari*':ti,ab,kw OR 'baylisascaris'/exp OR 'toxocar*':ti,ab,kw OR 'toxocara'/exp OR 'fasciol*':ti,ab,kw OR 'fasciola'/exp OR 'dirofilaria*':ti,ab,kw OR 'dirofilaria'/exp OR 'echinococc*':ti,ab,kw OR 'echinococcus'/exp OR 'taenia*':ti,ab,kw OR 'taenia'/exp OR 'trichin*':ti,ab,kw OR 'trichinella'/exp OR 'bovine spongiform encephalopathy':ti,ab,kw OR 'bovine spongiform encephalopathy'/exp OR 'babesi*':ti,ab,kw OR 'babesia'/exp OR 'cryptosporidi*':ti,ab,kw OR 'cryptosporidium'/exp OR 'giardi*':ti,ab,kw OR 'giardia'/exp OR 'toxoplasm*':ti,ab,kw OR 'toxoplasma'/exp OR 'leishmani*':ti,ab,kw OR 'leishmania'/exp OR 'lymphocytic choriomeningitis':ti,ab,kw OR 'lymphocytic choriomeningitis virus'/exp OR 'batai virus':ti,ab,kw OR 'batai virus'/exp OR 'bhanja virus':ti,ab,kw OR 'bhanja virus'/exp OR 'california encephalitis':ti,ab,kw OR 'california encephalitis'/exp OR 'crimean-congo hemorrhagic fever':ti,ab,kw OR 'crimean-congo hemorrhagic fever virus'/exp OR 'dobrava-belgrade':ti,ab,kw OR 'dobrava':ti,ab,kw OR 'dobrava-belgrade virus' OR 'erve virus':ti,ab,kw OR 'erve virus' OR 'puumala':ti,ab,kw OR 'puumala virus'/exp OR 'rift valley fever':ti,ab,kw OR 'rift valley fever virus'/exp OR 'seoul virus':ti,ab,kw OR 'seoul virus'/exp OR 'hanta*':ti,ab,kw OR 'hantavirus'/exp OR 'tahyna virus':ti,ab,kw OR 'tahyna virus' OR 'encephalitis':ti,ab,kw OR 'encephalitis virus'/exp OR 'louping ill':ti,ab,kw OR 'louping ill virus'/exp OR 'rocio':ti,ab,kw OR 'rocio virus'/exp OR 'wesselsbron':ti,ab,kw OR 'wesselsbron virus' OR 'west nile':ti,ab,kw OR 'west nile virus'/exp OR 'hepatitis e':ti,ab,kw OR 'hepatitis e virus'/exp OR 'dhori virus':ti,ab,kw OR 'dhori virus' OR 'batken virus':ti,ab,kw OR 'batken virus' OR 'influenza':ti,ab,kw OR 'influenza'/exp OR 'thogoto virus':ti,ab,kw OR 'thogoto virus'/exp OR 'thogotovirus':ti,ab,kw OR 'thogotovirus'/exp OR 'ljungan virus':ti,ab,kw OR 'ljungan virus'/exp OR 'cowpox':ti,ab,kw OR 'cowpox virus'/exp OR 'monkeypox':ti,ab,kw OR 'monkeypox virus'/exp OR 'orf virus':ti,ab,kw OR 'orf virus'/exp OR 'colorado tick fever':ti,ab,kw OR 'colorado tick fever virus'/exp OR 'eyach virus':ti,ab,kw OR 'eyach virus' OR 'tribec':ti,ab,kw OR 'tribec virus' OR 'lyssavirus':ti,ab,kw OR 'lyssavirus'/exp OR 'rabies':ti,ab,kw OR 'rabies virus'/exp OR 'barmah forest':ti,ab,kw OR 'barmah forest virus'/exp OR 'ross river':ti,ab,kw OR 'ross river virus'/exp OR 'sindbis':ti,ab,kw OR 'sindbis virus'/exp OR 'anisakis simplex*':ti,ab,kw OR 'anisakis simplex'/exp OR 'plague':ti,ab,kw OR 'plague'/exp OR 'typhus':ti,ab,kw OR 'typhus'/exp OR 'rat bite fever':ti,ab,kw OR 'rat bite fever'/exp OR 'rat-bite fever':ti,ab,kw OR 'rat-bite fever'/exp OR 'haverhill fever':ti,ab,kw OR 'haverhill fever'/exp OR 'anthrax':ti,ab,kw OR 'anthrax'/exp OR 'bacillus anthracis':ti,ab,kw OR 'bacillus anthracis'/exp OR 'botulism':ti,ab,kw OR 'botulism'/exp OR 'listeri*':ti,ab,kw OR 'listeria'/exp OR 'tuberculos*':ti,ab,kw OR 'tuberculosis'/exp OR 'q fever':ti,ab,kw OR 'q fever'/exp

Table S2 European countries (n=39) included in the literature search. Islands located far from mainland Europe (i.e., The Azores, Canary Islands, Faroe Islands and Madeira) were excluded from this review.

Albania	Estonia	Lithuania	Slovakia
Austria	Finland	Luxembourg	Slovenia
Belarus	France	Macedonia	Spain
Belgium	Germany	Moldova	Sweden
Bosnia and Herzegovina	Greece	Montenegro	Switzerland
Bulgaria	Hungary	Norway	The Netherlands
Croatia	Ireland	Poland	Turkey
Cyprus	Italy	Portugal	Ukraine
Czech Republic	Kosovo	Romania	United Kingdom
Denmark	Latvia	Serbia	

Table S3 Overview of all studied (potentially) zoonotic pathogens (bacteria, viruses, helminths, protozoa and fungi) in different wildlife species, showing the number of studies in which the pathogen was detected/the total number of studies performed. Detected pathogens are in bold. [a] host-pathogen combinations not studied in the Netherlands. [b] host-pathogen combinations studied in the Netherlands but without any positive results. - host-pathogen combination was not studied.

			Animals										
		**Order**	*Rodentia*					*Lago-morpha*	*Eulipotyphla*		*Carnivora*		
		**Family**	*Muridae*			*Cricetidae*	*Sciuridae*	*Leporidae*	*Erinaceidae*	*Talpidae*	*Mustelidae*	*Canidae*	
	**Genus**	**Species**	**Brown rat**	**House mouse**	**Wood mouse**	**Common vole**	**Red squirrel**	**European rabbit**	**European hedgehog**	**European mole**	**Stone marten**	**Red fox**	Number of animal species with the pathogen detected
	**BACTERIA**												
Bacterial pathogens	*Aerococcus*	*viridans*	-	-	-	-	0/1[a]	-	-	-	-	-	0[a]
	Anaplasma	spp	0/1[a]	-	0/1[a]	0/1[a]		-	-	-	0/1[a]	**1/4**[a]	1[a]
		phagocytophilum	**1/2**[a]	**1/4**[a]	**7/11**	**2/3**[a]	**3/4**[a]	-	**4/4**[a]	0/3[a]	**2/3**[b]	**14/18**	8
	Arcanobacterium	*haemolyticum*	-	-	-	-	-	-	**1/1**[a]	-	-	-	1[a]
	Bacillus	spp	**1/1**	-	-	-	**1/1**[a]	-	**1/1**[a]	-	-	-	3
		cereus	-	-	-	-	**1/1**[a]	-	-	-	-	-	1[a]
	Bacteroides	*pyogenes*	-	-	-	-	-	-	**1/1**[a]	-	-	-	1[a]
	Bartonella	spp	**6/7**[a]	0/1[a]	**7/11**[a]	**7/8**[a]	**2/2**[a]	**1/1**	**2/2**[a]	**1/1**[a]	0/2[a]	0/4[a]	7
		alsatica	-	-	-	-	-	**4/4**	-	-	-	-	1
		clarridgeiae	-	-	-	-	-	-	-	-	**1/1**[a]	-	1[a]
		grahamii	-	**1/1**[a]	**3/3**	-	**1/1**[a]	-	-	-	-	-	3
		henselae	**1/1**[a]	-	**1/1**[a]	-	-	-	-	-	-	-	2[a]
		melophagi	-	-	-	-	-	-	**1/1**[a]	-	-	-	1[a]
		rochalimae	-	-	-	-	-	-	-	-	-	**5/5**[a]	1[a]
		tribocorum	**3/3**	-	-	-	-	-	-	-	-	-	1
		vinsonii	-	-	**1/1**[a]	-	-	-	-	-	-	-	1[a]
		washoensis	-	-	-	-	**3/3**	-	**1/1**[a]	-	-	-	2
	Bordetella	*bronchiseptica*	-	-	-	-	-	**1/1**[a]	**1/1**[a]	-	-	-	2[a]

Table S3 (continued)

			Animals										
	**Genus**	**Species**	**Brown rat**	**House mouse**	**Wood mouse**	**Common vole**	**Red squirrel**	**European rabbit**	**European hedgehog**	**European mole**	**Stone marten**	**Red fox**	Number of animal species with the pathogen detected
Bacterial pathogens	*Borrelia*	spp	0/2[a]	**1/1[a]**	**2/6[a]**	**2/3[a]**	-	-	**2/2[a]**	0/1[a]	-	**2/4[a]**	5[a]
		afzelii	-	**1/2[a]**	**5/6**	**2/2[a]**	**5/5[a]**	-	**4/4[a]**	-	**2/2[b]**	**3/4[a]**	7
		bavariensis	-	-	-	-	-	-	**2/2[a]**	-	-	-	1[a]
		bissettiae	-	-	-	-	-	-	-	-	-	**1/1[a]**	1[a]
		burgdorferi s.l.	**2/3[a]**	**3/3[a]**	**13/16**	**7/7[a]**	**4/4[a]**	-	**2/2[a]**	0/2[a]	0/1[a]	**5/8[a]**	7
		burgdorferi s.s.	-	**1/2[a]**	**1/1[a]**	**1/1[a]**	**4/4[a]**	-	**1/1[a]**	-	-	-	5[a]
		garinii	-	0/2[a]	-	**2/2[a]**	**3/3[a]**	-	**2/2[a]**	-	-	**4/4[a]**	4[a]
		lusitaniae	-	0/1[a]	**1/1[a]**	-	-	-	-	-	-	**2/2[a]**	2[a]
		miyamotoi	-	-	**2/3**	0/1[a]	**1/1[a]**	-	0/1[a]	0/1[a]	0/1[a]	0/1[a]	2
		spielmanii	-	-	-	-	-	-	**3/3[a]**	-	-	**1/1[a]**	2[a]
	Brucella	spp	0/1	-	-	**1/1[a]**	-	-	-	-	-	**1/2[a]**	2
	Campylobacter	spp	**2/2**	**1/2**	0/1[a]	-	-	-	-	-	-	0/1[a]	2
		coli	-	**1/1**	-	-	-	-	-	-	-	-	1
		hyointestinalis	-	**1/1**	-	-	-	-	-	-	-	-	1
		jejuni	**1/1[a]**	**1/1**	-	-	**1/1[a]**	**1/1[a]**	**1/1**	-	-	-	5
	Chlamydia	spp	**1/1**	-	-	-	-	-	-	-	-	-	1
	Chlamydophila	spp	-	-	-	-	-	-	-	-	-	0/1[a]	0[a]
		psittaci	-	-	-	**1/1[a]**	-	-	-	-	-	-	1[a]
	Citrobacter	*freundii*	-	-	-	-	-	-	**2/2[a]**	-	-	-	1[a]
	Clostridium	*difficile*	**1/1**	**4/4**	**2/2**	**1/1**	-	-	-	-	-	-	4
	Coccobacillus	spp	-	-	-	-	**1/1[a]**	-	-	-	-	-	1[a]

Table S3 (continued)

Bacterial pathogens

		Animals										
**Genus**	**Species**	**Brown rat**	**House mouse**	**Wood mouse**	**Common vole**	**Red squirrel**	**European rabbit**	**European hedgehog**	**European mole**	**Stone marten**	**Red fox**	Number of animal species with the pathogen detected
Corynebacterium	spp	**1/1**	-	-	-	-	-	-	-	-	-	1
	confusum	-	-	-	-	-	-	**1/1**[a]	-	-	-	1[a]
	diphtheriae	-	-	-	-	-	-	-	-	-	**1/1**[a]	1[a]
	ulcerans	-	-	-	-	-	-	**3/3**[a]	-	-	-	1[a]
Coxiella	*burnetii*	**6/6**	**2/2**[a]	**5/8**[a]	**1/4**[a]	-	**4/5**[a]	-	0/2[a]	0/2[a]	**4/8**[a]	6
Dermatophilus	*congolensis*	-	-	-	-	**1/1**[a]	-	-	-	-	-	1[a]
Ehrlichia	spp	-	-	0/2[a]	0/1[a]	-	-	-	-	-	**2/2**	1
	canis	-	-	-	-	-	-	-	-	0/1[a]	**6/16**[a]	1[a]
Enterococcus	spp	-	-	**1/1**[a]	-	-	-	-	-	-	**2/3**[a]	2[a]
	spp AMR	-	-	**1/1**[a]	-	-	-	-	-	-	-	1[a]
	avium	-	-	-	-	-	-	**2/2**[a]	-	-	-	1[a]
	faecalis	-	-	-	-	**1/1**[a]	**1/1**[a]	**1/1**[a]	-	-	**2/2**[a]	4[a]
	faecium	-	-	-	-	**1/1**[a]	**1/1**[a]	-	-	-	**2/2**[a]	3[a]
	hirae	-	-	-	-	-	**1/1**[a]	**1/1**[a]	-	-	**2/2**[a]	3[a]
Escherichia (/Shigella)	spp	**1/2**	0/1[a]	-	-	-	-	-	-	**1/1**[a]	0/1[a]	2
	coli	-	0/1[a]	-	**1/1**[a]	**1/1**[a]	**2/2**[a]	**4/4**[a]	-	-	**2/2**[a]	5[a]
	coli AMR	**5/5**[a]	**1/1**[a]	**2/2**[a]	-	-	**3/4**[a]	**1/1**	-	**1/1**[a]	**7/8**[a]	7
Francisella	*tularensis*	**1/3**[a]	**1/1**[a]	**2/5**[a]	**6/10**[b]	**1/2**[a]	**3/3**[a]	-	0/1[a]	**2/2**[a]	**4/7**[a]	8
Helicobacter	spp	-	-	-	-	-	-	-	-	-	**2/2**[a]	1[a]
Klebsiella	spp	-	-	-	-	-	-	**1/1**[a]	-	**1/1**[a]	-	2[a]
	pneumoniae	-	-	-	-	-	-	**2/2**[a]	-	-	-	1[a]
Lactococcus	*garvieae*	-	-	-	-	-	-	**1/1**[a]	-	-	-	1[a]

Table S3 (continued)

	Genus	Species	Brown rat	House mouse	Wood mouse	Common vole	Red squirrel	European rabbit	European hedgehog	European mole	Stone marten	Red fox	Number of animal species with the pathogen detected
			Animals										
Bacterial pathogens	*Leptospira*	spp	**13/14**[a]	**4/4**[a]	**9/12**[b]	**14/14**[a]	-	-	**3/3**	0/3[a]	-	**2/2**[a]	6
		borgpetersenii	**1/1**[a]	**2/2**[a]	**1/1**[a]	-	-	-	**4/4**	-	-	-	4
		interrogans	**13/13**	**4/5**[a]	**4/5**[a]	**3/3**	-	-	**7/7**	-	**2/2**[a]	**9/10**[a]	7
		kirschneri	0/1	**1/2**[a]	-	-	-	-	-	-	-	-	1
	Listeria	spp	**1/1**[a]	-	-	-	-	-	-	-	-	**1/1**[a]	2[a]
		ivanovii	-	-	-	-	-	-	-	-	-	**1/1**[a]	1[a]
		monocytogenes	-	-	-	-	-	-	**1/1**[a]	-	**1/3**[a]	**2/4**[a]	3[a]
	Morganella	*morganii*	-	-	-	-	-	-	**2/2**[a]	-	-	-	1[a]
	Mycobacterium	spp	**1/1**	**1/1**[a]	-	**1/1**[a]	**1/2**[a]	-	**1/1**[a]	**1/1**[a]	**1/1**[a]	**1/2**[a]	8
		avium	-	**1/1**[a]	**1/1**[a]	-	-	**8/8**[a]	**1/1**[a]	**1/1**[a]	**2/3**[a]	**2/3**[a]	7[a]
		bovis	**1/1**[a]	-	**1/1**[a]	-	-	0/1[a]	0/1[a]	-	**1/1**[a]	**9/9**[a]	4[a]
		caprae	-	-	-	-	-	-	-	-	-	**2/3**[a]	1[a]
		leprae	-	-	-	-	**2/4**[b]	-	-	-	-	-	1
		lepromatosis	-	-	-	-	**1/3**[b]	-	-	-	-	-	1
	Mycoplasma	spp	-	-	-	**1/1**[a]	-	-	-	-	-	-	1[a]
		haemofelis	**1/1**[a]	**1/1**[a]	-	-	-	-	-	-	-	-	2[a]
		pulmonis	**1/1**[a]	-	-	-	-	-	-	-	-	-	1[a]
	Neoehrlichia	spp	-	-	-	-	-	-	-	-	-	**4/4**[a]	1[a]
		mikurensis	**1/1**[a]	0/1[a]	**2/2**	**1/2**[a]	0/1[a]	-	-	-	**1/1**[b]	0/4[a]	4
	Pantoea	*agglomerans*	-	-	-	-	**1/1**[a]	-	-	-	-	-	1[a]
	Pasteurella	spp	**1/1**[a]	-	-	-	-	-	-	-	**1/1**[a]	-	2[a]
		canis	-	-	-	-	-	-	-	-	-	**1/1**[a]	1[a]
		multocida	0/1[a]	-	-	-	-	-	**1/1**[a]	-	-	-	1[a]
		pneumotropica	**1/1**[a]	-	-	-	-	-	-	-	-	-	1[a]

Table S3 (continued)

			Animals										
	**Genus**	**Species**	**Brown rat**	**House mouse**	**Wood mouse**	**Common vole**	**Red squirrel**	**European rabbit**	**European hedgehog**	**European mole**	**Stone marten**	**Red fox**	Number of animal species with the pathogen detected
Bacterial pathogens	*Proteus*	spp	**1/1**										1
		mirabilis							**2/2[a]**				1[a]
	Pseudomonas	spp	**1/1[a]**										1[a]
	Rickettsia	spp	**2/2[a]**	0/1[a]	**1/5 [b]**	**4/8[a]**			**1/2[a]**	**1/2[a]**	0/1[a]	0/10[a]	5
		conorii	**1/1[a]**					**1/1[a]**				**1/1[a]**	3[a]
		helvetica	0/1[a]	**1/1[a]**						0/1[a]	**1/1[a]**		2[a]
		massiliae										**1/1[a]**	1[a]
		prowazekii						0/1[a]					0[a]
		slovaca						**1/1[a]**				**1/1[a]**	2[a]
		typhi	**4/4[a]**	**1/1[a]**	**1/1[a]**			0/1[a]				**1/1[a]**	4[a]
	Salmonella	spp	**2/5 [b]**	0/4	0/1	0/1[a]			**1/1**			**1/3[a]**	3
		enterica		**2/2**				**1/1[a]**	**7/7**		**1/1[a]**	**8/8[a]**	5
	Spiroplasma	*ixodetes*			0/1								0
	Staphylococcus	spp	**2/2**								**1/1[a]**	**1/1[a]**	3
		aureus	**1/1**		0/1[a]	**1/1[a]**	**5/5[a]**		**1/1[a]**		**1/1[a]**	**1/1[a]**	6
		aureus (MRSA)	**1/2[b]**	**1/2[b]**	**1/2[b]**			**2/2[a]**	**8/8[a]**			0/2[a]	5
		lentus							**1/1[a]**			**1/1[a]**	2[a]
		delphini									**1/1[a]**	**1/1[a]**	2[a]
		equorum										**1/1[a]**	1[a]
		hycus					0/1[a]						0[a]
		pseudintermedius									**1/1[a]**	**1/1[a]**	2[a]
		sciuri					**1/1[a]**		**1/1[a]**			**1/1[a]**	3[a]
		simulans							**1/1[a]**				1[a]

Table S3 (continued)

	Genus	Species	Brown rat	House mouse	Wood mouse	Common vole	Red squirrel	European rabbit	European hedgehog	European mole	Stone marten	Red fox	Number of animal species with the pathogen detected
			Animals										
Bacterial pathogens	*Streptobacillus*	*moniliformis*	-	-	-	-	-	-	-	**1/1[a]**	-	-	1[a]
	Streptococcus	spp	**1/1**	-	-	-	-	-	-	-	**1/1[a]**	-	2
		canis	-	-	-	-	-	-	**1/1[a]**	-	-	**2/2[a]**	2[a]
		gallinaceus	-	-	-	-	-	-	**1/1[a]**	-	-	-	1[a]
		pyogenes	-	-	-	-	-	-	**2/2[a]**	-	-	-	1[a]
		suis	-	-	-	-	-	**1/1[a]**	-	-	-	-	1[a]
		thoraltensis	-	-	-	-	-	-	**1/1[a]**	-	-	-	1[a]
	Trueperella	*pyogenes*	-	-	-	-	-	-	**1/1[a]**	-	-	-	1[a]
	Vagococcus	*fluvialis*	-	-	-	-	-	-	**1/1[a]**	-	-	-	1[a]
		lutrae	-	-	-	-	-	-	**1/1[a]**	-	-	-	1[a]
	Yersinia	spp	**2/2[a]**	-	-	**1/1[a]**	-	-	-	-	-	**1/1[a]**	3[a]
		enterocolitica	**5/5[a]**	**2/3[a]**	**3/4[a]**	**2/2[a]**	-	-	-	-	**3/3[a]**	**6/7[a]**	6[a]
		pseudotuberculosis	**1/1[a]**	**2/2[a]**	-	-	-	-	-	-	**1/1[a]**	**1/1[a]**	4[a]
	Nr of bacterial pathogens detected		39	27	27	22	24	17	51	5[a]	23[a]	51	
	**VIRUSES**												
Viral pathogens	*Alphainfluenza-virus*	*Influenza A virus*	0/1[a]	-	-	-	-	-	0/1[a]	-	**1/2[a]**	**3/4**	2
	Alphavirus	*Sindbis virus*	-	-	-	-	-	-	**1/1[a]**	-	-	-	1[a]
	Aphthovirus	*Foot and mouth disease virus*	-	-	-	-	-	-	**1/1[a]**	-	-	-	1[a]
	Betacoronavirus	*SARS-CoV-2*	0/1[a]	0/1[a]	0/2[a]	0/1[a]	-	-	-	-	**1/2**	0/2[a]	1
	Cardiovirus	*Encephalo-myocarditis virus*	0/1[a]	-	-	-	-	-	-	-	-	-	0[a]
	Coltivirus	*Eyach coltivirus*	-	-	0/1	-	-	0/1[a]	-	-	-	-	0

Table S3 (continued)

			Animals										
	**Genus**	**Species**	**Brown rat**	**House mouse**	**Wood mouse**	**Common vole**	**Red squirrel**	**European rabbit**	**European hedgehog**	**European mole**	**Stone marten**	**Red fox**	Number of animal species with the pathogen detected
Viral pathogens	*Flavivirus*	spp	0/1[a]	-	0/1[a]	-	-	-	-	-	-	-	0[a]
		Tick-borne encephalitis virus	-	0/1[a]	**2/4[b]**	**4/4**	0/1[a]	-	**1/1[a]**	**2/2[a]**	0/1[a]	**1/2[a]**	5
		Usutu virus	0/1[a]	-	-	-	-	-	-	-	-	-	0[a]
		West nile virus	0/1[a]	-	0/1[a]	-	-	-	**1/1[a]**	-	-	**1/2[a]**	2[a]
	Kobuvirus	spp	**1/1[a]**	-	-	-	-	-	-	-	-	**1/1[a]**	2[a]
	Lyssavirus	*European bat lyssavirus*	-	-	-	-	-	-	-	-	**1/1[a]**	-	1[a]
		Lyssavirus rabies	-	-	-	1/2[a]	-	-	-	-	**1/2[a]**	**3/8[a]**	3[a]
	Mammarena-virus	*Lymphocytic choriomeningitis mammarenavirus*	-	**4/4[a]**	**5/6[a]**	**3/3[a]**	-	-	-	-	-	**1/1[a]**	4[a]
	Norovirus	spp	**4/4[a]**	-	-	-	-	-	-	-	-	-	1[a]
	Orthobornavirus	*Borna disease virus*	-	-	-	-	-	-	-	-	0/1[a]	**2/4[a]**	1[a]
	Orthohantavirus	spp	**4/5[a]**	**2/2[a]**	**3/6[a]**	**5/7[a]**	-	-	-	-	-	**2/2[a]**	5[a]
		Dobrava-Belgrade orthohantavirus	-	0/1[a]	**2/8[a]**	**2/2[a]**	-	-	-	-	-	-	2[a]
		Puumala orthohantavirus	-	**2/3[a]**	**3/10[b]**	**1/3[a]**	-	-	-	-	-	**1/1** [a]	4
		Seoul orthohantavirus	**9/10**	0/1[a]	-	-	-	-	-	-	-	-	1
		Tula orthohantavirus	-	0/2[a]	**2/6[b]**	**25/26**	-	-	-	-	-	-	2
	Orthohepevirus	spp	**1/1[a]**			**2/2[a]**	-	-	-	-	-	-	2[a]
		Hepatitis E virus	**10/12[a]**	0/1[a]	0/1[a]	-	-	**5/6**	-	-	-	**2/4[a]**	3

Table S3 (continued)

	Animals											
	**Genus**	**Species**	**Brown rat**	**House mouse**	**Wood mouse**	**Common vole**	**Red squirrel**	**European rabbit**	**European hedgehog**	**European mole**	**Stone marten**	**Red fox**	Number of animal species with the pathogen detected
Viral pathogens	*Orthonairovirus*	*Crimean-congo haemorrhagic fever virus*	-	-	0/1[a]	-	-	0/1[a]	-	-	-	-	0[a]
	Orthopoxvirus	spp	0/1[a]	**1/1[a]**	**4/7[a]**	**2/2[a]**	-	-	-	-	-	-	3[a]
		Cowpoxvirus	**3/4**	**1/1[a]**	**3/3[a]**	**3/3[a]**	-	-	-	-	-	-	4
	Parechovirus	*Ljungan virus*	-	**1/1[a]**	**1/1[a]**	-	**1/1[a]**	-	-	-	-	-	3[a]
	Phlebovirus	spp	-	-	-	-	-	-	-	-	-	**1/1[a]**	1[a]
	Rotavirus	spp	**2/2[a]**	-	-	-	0/1[a]	-	-	-	-	**2/2[a]**	2[a]
	Varicellovirus	*Pseudorabies virus*	-	-	-	-	-	-	-	-	-	**1/1[a]**	1[a]
		Nr of viral pathogens detected	8	6[a]	9	10	1[a]	1	4[a]	1[a]	4	13	
	**HELMINTHS**												
Helminth pathogens	*Alaria*	spp	-	-	-	-	-	-	-	-	**1/1[a]**	**1/1[a]**	2[a]
		alata	**1/1[a]**	-	-	-	-	-	-	-	-	**21/21**	2
	Ancylostoma	spp	-	-	-	-	-	-	-	-	-	**1/1[a]**	1[a]
		caninum	-	-	-	-	-	-	-	-	-	**7/7[a]**	1[a]
	Angiostrongylus	spp		-	-	-	-	-	-	-	**1/2[a]**	-	1[a]
		cantonensis	**1/1[a]**	-	-	-	-	-	-	-	-	-	1[a]
	Brachylaima	spp	-	-	**3/3[a]**	-	-	-	-	-	-	-	1[a]
	Capillaria	spp	**2/2[a]**	-	-	-	-	-	**5/5[a]**	-	**2/3[a]**	**8/8**	4
		aerophila	-	-	-	-	-	-	-	-	-	**26/26**	1
		hepatica	**7/7[a]**	0/1[a]	**2/2[a]**	**1/1[a]**	**2/2[a]**	-	-	-	-	**1/1[a]**	5[a]
	Dicrocoelium	*dendriticum*	-	-	-	-	-	-	-	-	-	**1/1[a]**	1[a]
	Dioctophyma	*renale*	-	-	-	-	-	-	-	-	-	**1/1[a]**	1[a]
	Dipetalonema	spp	-	-	-	-	-	-	-	-	-	0/1[a]	0[a]

Table S3 (continued)

			Animals										
	**Genus**	**Species**	**Brown rat**	**House mouse**	**Wood mouse**	**Common vole**	**Red squirrel**	**European rabbit**	**European hedgehog**	**European mole**	**Stone marten**	**Red fox**	Number of animal species with the pathogen detected
Helminth pathogens	*Diphyllobothrium*	spp	-	-	-	-	-	-	-	-	-	**3/3**[a]	1[a]
	Dipylidium	spp	-	-	-	-	-	-	-	-	-	**2/2**[a]	1[a]
		caninum	-	-	-	-	-	-	-	-	-	**18/18**[a]	1[a]
	Dirofilaria	spp	-	-	-	-	-	-	-	-	-	**1/2**[a]	1[a]
		immitis	-	-	-	-	-	-	-	-	0/1[a]	**13/16**[a]	1[a]
		repens	-	-	-	-	-	-	-	-	**1/1**[a]	**4/6**[a]	2[a]
	Echinochasmus	*perfoliatus*	-	-	-	-	-	-	-	-	-	**3/3**[a]	1[a]
	Echinococcus	spp	0/1[a]	-	-	-	-	-	-	-	-	**1/3**[b]	1
		granulosus	-	-	-	-	-	-	-	-	-	**2/6**[a]	1[a]
		multilocularis	**1/1**[a]	**1/1**[a]	0/1[a]	**3/3**[a]	-	-	-	-	0/4[a]	**70/75**	4
	Fasciola	*hepatica*	0/1[a]	-	-	-	-	**3/3**	-	-	-		1
	Heterophyes	*heterophyes*	-	-	-	-	-	-	-	-	-	**1/1**[a]	1[a]
	Hymenolepis	spp	-	-	**3/3**[a]	-	-	-	-	-	-	**2/2**	2
		diminuta	**7/7**	**1/1**[a]	0/1	-	-	-	-	-	-	**1/1**[a]	3
		microstoma	**1/1**[a]	-	-	-	-	-	-	-	-		1[a]
		nana	**7/7**	0/1[a]	-	-	-	-	-	-	-	**1/1**[a]	2
	Mesocestoides	spp	-	-	**3/3**[a]	-	-	-	-	-	**1/1**[a]	**22/23**[b]	3
		lineatus	-	-	-	-	-	-	-	-	-	**3/3**[a]	1[a]
	Metagonimus	*yokogawai*	-	-	-	-	-	-	-	-	-	**1/1**[a]	1[a]
	Metorchis	*bilis*	-	-	-	-	-	-	-	-	-	**4/4**[a]	1[a]
	Moniliformis	*moniliformis*	**1/1**[a]	-	-	-	-	-	-	-	-	-	1[a]
	Onchocerca	spp	-	-	-	-	-	-	-	-	-	0/1[a]	0[a]
	Opisthorchis	*felineus*	-	-	-	-	-	-	-	-	-	**4/4**[a]	1[a]

Table S3 (continued)

Helminth pathogens

		Animals										
**Genus**	**Species**	**Brown rat**	**House mouse**	**Wood mouse**	**Common vole**	**Red squirrel**	**European rabbit**	**European hedgehog**	**European mole**	**Stone marten**	**Red fox**	Number of animal species with the pathogen detected
Pelodera	*strongyloides*	-	-	**1/1**[a]	-	-	-	-	-	-	-	1[a]
Physaloptera	spp	-	-	-	-	-	-	-	-	-	**2/2**[a]	1[a]
Plagiorchis	*muris*	-	-	**1/1**[a]	-	-	-	-	-	-	-	1[a]
Pseudamphistomum	*truncatum*	-	-	-	-	-	-	-	-	-	**6/6**[a]	1[a]
Schistosoma	spp	-	0/1[a]	-	-	-	-	-	-	-	-	0[a]
Spirometra	spp	-	-	-	-	-	-	-	-	-	**1/1**[a]	1[a]
	erinacei	-	-	-	-	-	-	-	-	-	**1/1**[a]	1[a]
Strongyloides	spp	**1/1**	-	-	-	-	-	-	-	**1/1**[a]	**4/4**	3
Taenia	spp	-	-	-	-	-	-	-	-	**1/1**[a]	**20/20**	2
	crassiceps	-	-	-	**1/1**[a]	-	-	-	-	**1/1**[a]	**14/14**	3
	martis	-	-	**4/4**[a]	-	**1/1**[a]	-	-	-	**1/1**[a]	**1/1**[a]	4[a]
	multiceps	-	-	-	-	-	-	-	-	-	**3/3**[a]	1[a]
	serialis	-	-	-	-	-	-	-	-	-	**2/2**[a]	1[a]
	taeniaeformis	**4/4**[a]	0/1[a]	**6/6**[a]	**2/2**[a]	-	-	-	-	-	**6/6**[a]	4[a]
Thelazia	*callipaeda*	-	-	-	-	-	**1/1**[a]	-	-	**3/3**[a]	**5/6**[a]	3[a]
Toxascaris	*leonina*	-	-	-	-	-	-	-	-	**1/3**[a]	**27/28**	2
Toxocara	spp	0/1[a]	**1/1**[a]	-	-	-	-	-	-	**1/2**[a]	**5/5**[a]	3[a]
	canis	-	-	-	-	-	-	-	-	0/1[a]	**42/42**	1
	cati	**1/1**[a]	-	-	-	-	-	-	-	0/2[a]	**2/4**[a]	2[a]
Trichinella	spp	**4/7**[b]	-	-	-	-	-	0/1[a]	-	**4/5**[a]	**32/39**	3
	britovi	0/1[a]	-	-	-	-	-	-	-	**3/3**[a]	**31/31**[a]	2[a]
	nativa	0/1[a]	-	-	-	-	-	-	-	-	**11/12**[a]	1[a]
	pseudospiralis	**1/2**[a]	-	-	-	-	-	-	-	-	**7/10**[a]	2[a]
	spiralis	**3/4**[a]	-	-	-	-	-	-	-	-	**27/28**[a]	2[a]

Table S3 (continued)

	Animals												
	**Genus**	**Species**	**Brown rat**	**House mouse**	**Wood mouse**	**Common vole**	**Red squirrel**	**European rabbit**	**European hedgehog**	**European mole**	**Stone marten**	**Red fox**	Number of animal species with the pathogen detected
Helminth pathogens	*Trichostrongylus*	spp	-	-	-	-	-	-	-	-	-	**1/1[a]**	1[a]
	Trichuris	spp	-	-	-	-	-	-	-	-	0/1[a]	0/1[a]	0[a]
		vulpis	-	-	-	-	-	-	-	-	-	**16/16**	1
	Uncinaria	spp	-	-	-	-	-	-	-	-	**1/1[a]**	-	1[a]
		stenocephala	-	-	-	-	-	-	-	-	0/1[a]	**32/32**	1
	Nr of helminth pathogens det.		15	3[a]	8	4[a]	2[a]	2	1[a]	0[a]	15[a]	52	
	**PROTOZOA**												
Protozoal pathogens	*Babesia*	spp	0/3[a]	0/1[a]	**2/3**	0/1[a]	0/1[a]	-	0/1[a]	**1/1[a]**	0/2[a]	**9/11**	3
		microti	-	**1/1[a]**	**2/4**	**6/6[a]**	-	-	-	-	-	0/1[a]	3
	Blastocystis	spp	**1/2[a]**	-	-	-	**1/2[a]**	**1/1[a]**	-	-	-	-	3[a]
	Cryptosporidium	spp	**2/5[a]**	**1/1[a]**	0/2[a]	**2/2[a]**	**1/3[a]**	-	**2/2**	-	0/2[a]	**6/7[a]**	6
		andersoni	**1/1[a]**	-	-	-	-	**1/1[a]**	-	-	-	**1/1[a]**	3[a]
		canis	-	-	-	-	-	-	-	-	-	**2/2[a]**	1[a]
		chipmunk	-	-	-	-	**2/2[a]**	-	-	-	-	-	1[a]
		cuniculus	-	-	-	-	-	**1/1 [a]**	-	-	-	-	1[a]
		felis	-	-	-	-	-	-	-	-	-	**1/1[a]**	1[a]
		hominis	-	-	-	-	-	-	-	-	-	**1/1[a]**	1[a]
		muris	**1/1[a]**	**1/1[a]**	**2/2[a]**	-	-	-	0/1[a]	-	-	-	3[a]
		occultus	**1/1[a]**	-	-	-	-	-	-	-	-	-	1[a]
		parvum	**2/2[a]**	**1/1[a]**	**2/2[a]**	**1/1[a]**	**1/1[a]**	-	**3/3[a]**	-	-	**3/3[a]**	7[a]
		suis	-	-	-	-	-	-	-	-	-	**1/1[a]**	1[a]
		tyzzeri	-	-	-	-	-	-	-	-	-	**1/1[a]**	1[a]
		ubiquitum	-	-	-	-	-	-	-	-	-	**2/2[a]**	1[a]
	Dientamoeba	*fragilis*	**1/1[a]**	-	-	-	-	-	-	-	-	-	1[a]

Table S3 (continued)

			Animals										
	**Genus**	**Species**	**Brown rat**	**House mouse**	**Wood mouse**	**Common vole**	**Red squirrel**	**European rabbit**	**European hedgehog**	**European mole**	**Stone marten**	**Red fox**	Number of animal species with the pathogen detected
Protozoal pathogens	*Entamoeba*	spp	0/1[a]	-	-	-	0/1[a]	-	-	-	-	-	0[a]
	Giardia	spp	0/1[a]	-	-	**2/2[a]**	0/1[a]	-	**1/1**	-	**1/2[a]**	**3/4[a]**	4
		duodenalis	**1/1[a]**	-	-	-	0/1[a]	**1/1[a]**	-	-		**4/4[a]**	3[a]
	Leishmania	spp	**3/3[a]**	**2/2[a]**	0/1[a]	-	0/1[a]	**1/1[a]**	-	-	**1/2[a]**	**1/5[a]**	5[a]
		infantum	**5/5[a]**	**3/3[a]**	**2/3[a]**	-	**1/1[a]**	**9/9[a]**	**3/4[a]**	-	**4/4[a]**	**13/15[a]**	8[a]
	Neospora	*caninum*	**2/3[b]**	**3/6**	-	**2/2**	-	-	-	-	0/2[a]	**11/17[a]**	4
	Sarcocystis	spp	0/1[a]	0/1[a]	0/1[a]	-	-	-	-	-	**1/1[a]**	**2/2[a]**	2[a]
	Toxoplasma	spp	**1/1[a]**	-	-	-	-	**1/1[a]**	-	-	-	-	2[a]
		gondii	**8/9**	**11/13**	**5/9**	**3/4**	**5/5**	**5/5[a]**	**1/1[a]**	**3/3**	**4/5[a]**	**28/32[a]**	10
	Trypanosoma	spp	**1/1[a]**	0/3	**2/2[a]**	**1/1[a]**	-	**1/1[a]**	-	-	-	-	4
		lewisi	**3/3[a]**	0/1	-	-	-	-	-	-	-	-	1
	Nr of protozoal pathogens detected		15	8	7	7	6	9[a]	5	2	5[a]	17	
	**FUNGI**												
Fungal pathogens	*Aspergillus*	spp	-	-	-	-	-	-	-	-	**1/1[a]**	-	1[a]
		fumigatus	-	-	-	**1/1[a]**	-	-	-	-	-	-	1[a]
	Cryptococcus	*neoformans*	-	-	-	-	-	-	-	-	-	**1/1[a]**	1[a]
	Emmonsia	*crescens*	-	-	-	**1/1[a]**	-	-	-	**1/1[a]**	-	-	2[a]
	Encephalitozoon	*cuniculi*	-	**2/3[a]**	**2/2[a]**	**1/1[a]**	-	0/2[a]	-	-	**1/2[a]**	**1/4[a]**	5[a]
		hellem	-	**1/1[a]**	-	-	-	0/1[a]	-	-	-	-	1[a]
		intestinalis	-	**1/1[a]**	-	-	-	**2/2[a]**	-	-	-	**1/1[a]**	3[a]
	Enterocytozoon	*bieneusi*	-	**2/2[a]**	-	-	0/1[a]	**1/2[a]**	-	-	**2/2[a]**	**2/3[a]**	4[a]
	Histoplasma	*capsulatum*	-	-	-	-	-	-	**1/1[a]**	-	0/1[a]	-	1[a]
	Microsporum	*canis*	-	-	-	-	-	-	**1/1[a]**	-	-	-	1[a]

Table S3 (continued)

		Animals										
**Fungal pathogens**												
**Genus**	**Species**	**Brown rat**	**House mouse**	**Wood mouse**	**Common vole**	**Red squirrel**	**European rabbit**	**European hedgehog**	**European mole**	**Stone marten**	**Red fox**	Number of animal species with the pathogen detected
Nannizzia	*fulva*							**1/1[a]**				1[a]
	gypsea							**1/1[a]**				1[a]
	nana							**1/1[a]**				1[a]
Penicillium	spp				**1/1[a]**							1[a]
Scopulariopsis	*brevicaulis*				**1/1[a]**							1[a]
Trichophyton	*benhamiae*							**1/1[a]**				1[a]
	mentagrophytes		**1/1[a]**					**4/4[a]**				2[a]
Nr of fungal pathogens detected		0[a]	5[a]	1[a]	5[a]	0[a]	2[a]	7[a]	1[a]	3[a]	4[a]	

[a]: Host-pathogen combinations not studied in the Netherlands.

[b]: Host-pathogen combinations studied in the Netherlands but without any positive results.

Table S4 Coverage percentages per pathogen group, animal species and in total. The coverage percentage was calculated by dividing the number of studied host-pathogen combinations by the total number of host-pathogen combinations, multiplied by 100. HPCs = host-pathogen combinations. Coverage percentages >50% are highlighted in bold.

		**Brown rat**	**House mouse**	**Wood mouse**	**Common vole**	**Red squirrel**	**European rabbit**	**European hedgehog**	**European mole**	**Stone marten**	**Red fox**	**Total**
**Bacteria**	# studied HPCs	45	35	33	26	27	20	53	13	30	59	341/1210
	Coverage %	37%	29%	27%	21%	22%	17%	44%	11%	25%	49%	28%
**Viruses**	# studied HPCs	15	12	15	11	3	3	5	1	6	14	85/300
	Coverage %	**50%**	40%	**50%**	37%	10%	10%	17%	3%	20%	47%	28%
**Helminths**	# studied HPCs	20	7	10	4	2	2	2	0	21	55	123/650
	Coverage %	31%	11%	15%	6%	3%	3%	3%	0%	32%	**85%**	19%
**Protozoa**	# studied HPCs	19	12	10	8	11	9	7	2	8	18	104/280
	Coverage %	**68%**	43%	36%	29%	39%	32%	25%	7%	29%	**64%**	37%
**Fungi**	# studied HPCs	0	5	1	5	1	4	7	1	4	4	32/170
	Coverage %	0%	29%	6%	29%	6%	24%	41%	6%	24%	24%	19%
**Total**	# studied HPCs	99	71	69	54	44	38	74	17	69	150	685/2610
	Coverage %	38%	27%	26%	21%	17%	15%	28%	7%	26%	**57%**	26%

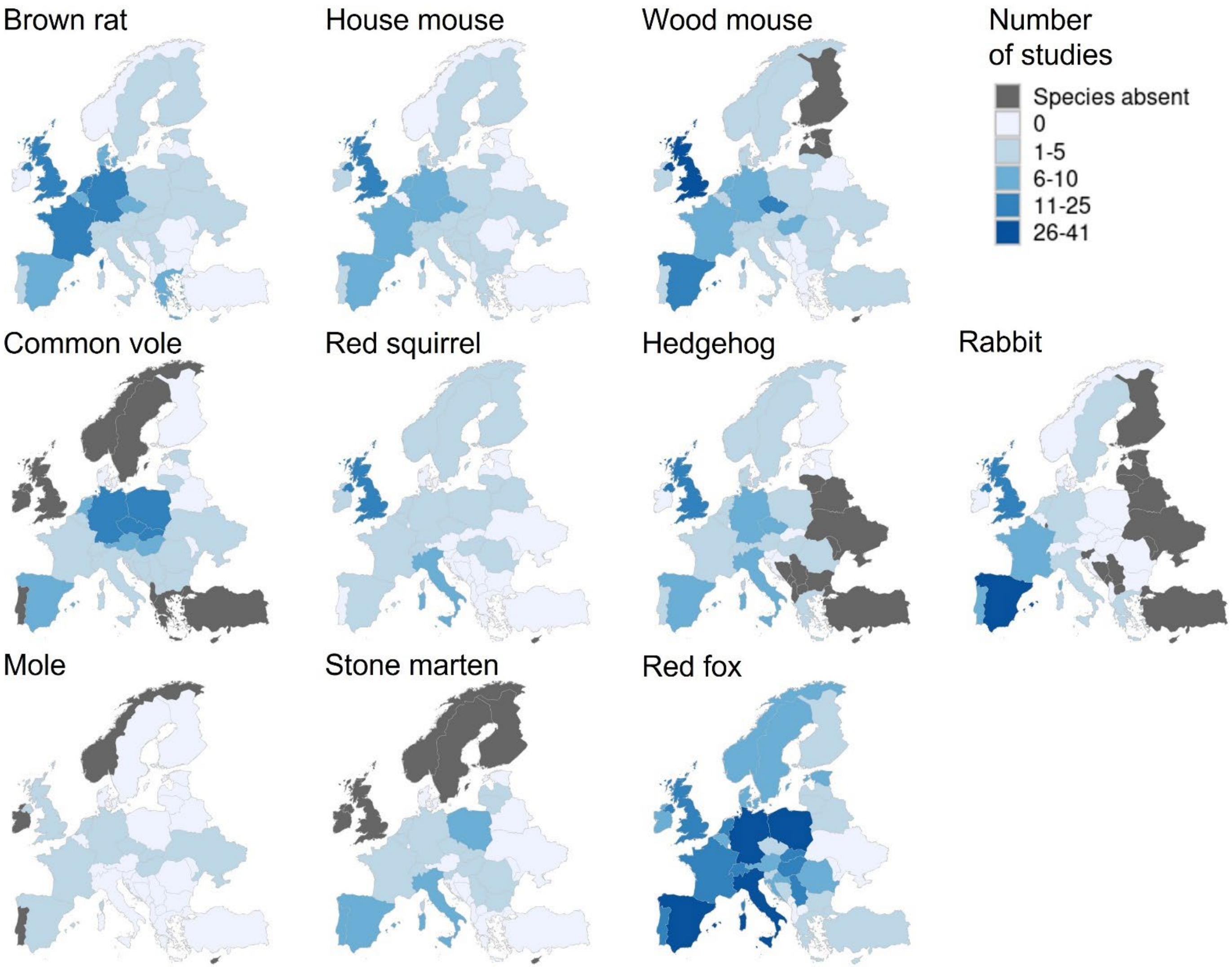

Figure S1 Number of studies conducted per animal species per country. 'Species absent' indicates when an animal species does not occur in a country. Animal distributions are taken from the IUCN Red List Version 2022-2 (IUCN, 2023).

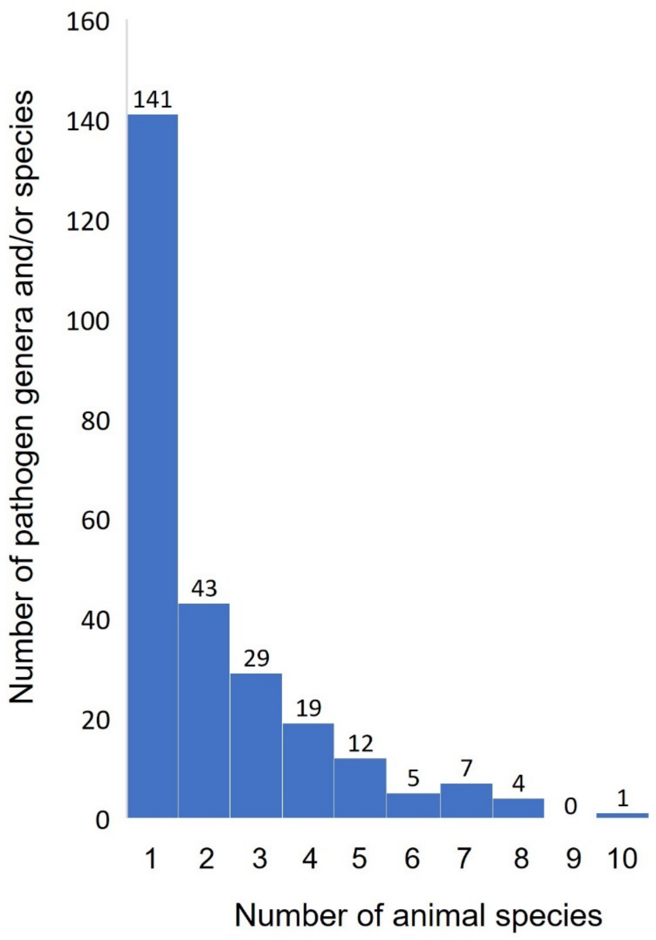

Figure S2 Summary of Table S3 (right hand column, 261 sp./spp.), indicating the number of pathogens (sp./spp.) detected in one or multiple animal species. For example, there were 141 pathogens that were only detected in a single animal species, whereas there was one pathogen that was detected in all 10 animal species.

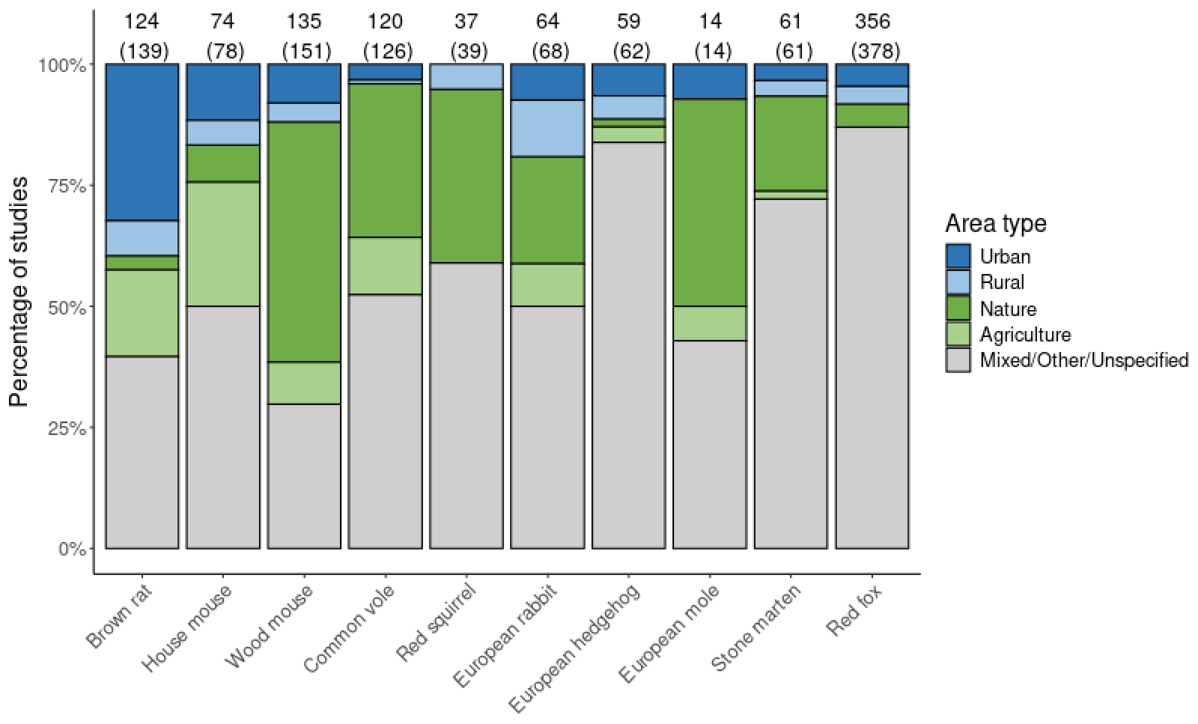

Figure S3 Relative percentage of studies performed in a specific habitat type (e.g., urban, rural, nature, agriculture or mixed/other/unspecified) per animal species. Mixed habitats included >1 habitat type but were not specified separately. Above the bars, the total number of studies per animal species is shown, with in brackets the total number of area types described in these studies.

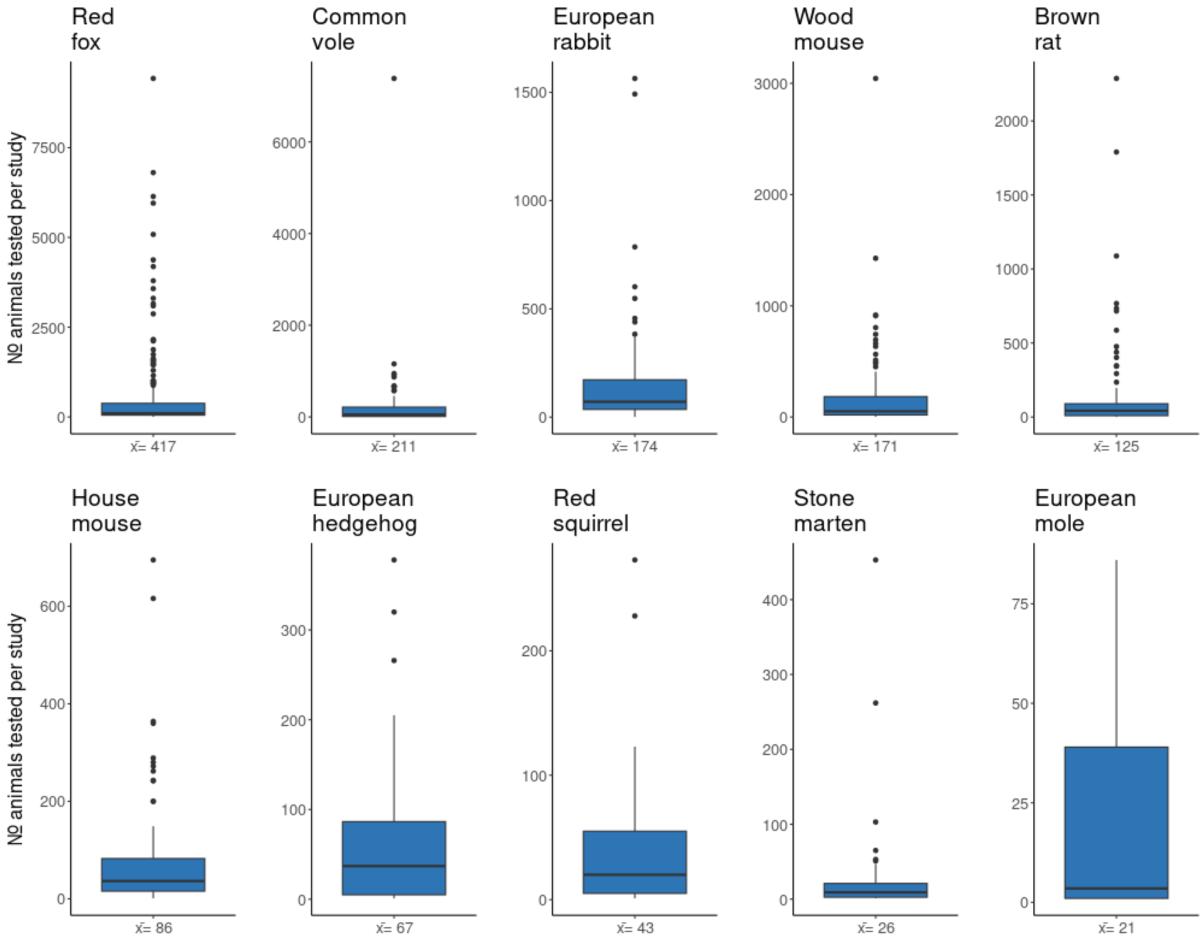

Figure S4 Number of animals tested per study, per animal species. x̄ shows the mean number of animals tested per study. The y-axes have different scales.
